# Comprehensive mapping of molecular cytogenetic markers in pitaya (*Hylocereus undatus*) and related species

**DOI:** 10.3389/fpls.2024.1493776

**Published:** 2024-12-06

**Authors:** Arrashid Harun, Shipeng Song, Xixi You, Hui Liu, Xiaopeng Wen, Zhongming Fang, Zhihao Cheng, Chunli Chen

**Affiliations:** ^1^ Key Laboratory of Plant Resource Conservation and Germplasm Innovation in Mountainous Region (Ministry of Education), Institute of Agro-bioengineering, College of Life Science, Guizhou University, Guiyang, Guizhou, China; ^2^ National Key Laboratory for Germplasm Innovation and Utilization for Fruit and Vegetable Horticultural Crops Hubei Hongshan Laboratory, Huazhong Agricultural University, Wuhan, Hubei, China; ^3^ College of Life Science and Technology, Huazhong Agricultural University, Wuhan, Hubei, China; ^4^ Sanya Research Institute, National Key Laboratory for Tropical Crop Breeding, Chinese Academy of Tropical Agricultural Sciences, Sanya, Hainan, China

**Keywords:** pitaya, cacti, oligo, rDNA, tandem repeat, mapping, karyotype, cytogenetic marker

## Abstract

Pitaya (*Hylocereus undatus*; 2n=22) is an important fruit crop from the *Cactaceae* family, originally domesticated in Mexico and the USA, and is now widely cultivated for its nutritional benefits. It is characterized by its distinctive triangular-shaped stems and large, showy flowers, thriving in arid and semi-arid environments, particularly in hot, dry climates. However, systematic chromosomal studies, including chromosomal mapping of cytogenetic markers in pitaya, are limited, presenting challenges for its cytogenetic improvement. To address this issue, we designed oligo-barcodes specific to thirty-three chromosome regions based on the pitaya reference genome and applied them to both pitaya and cactus (*Selenicerus grandifloras*; 2n=22) for oligo-barcodes mapping, karyotyping, and chromosome identification. We utilized FISH technology, employing oligo, rDNA, and tandem repeat probes for chromosomal mapping, identification, and karyotyping of pitaya and related species. We successfully localized oligo-barcodes on eleven pairs of chromosomes in both pitaya and cactus, demonstrating the effectiveness of the synthesized oligo-barcodes. We used two ribosomal DNA (rDNA) probes (45S and 5S) and two tandem repeat probes (GTR11 and STR3) in pitaya (both diploid and tetraploid) and two other *Cactaceae* species (*S. grandifloras* and *Opuntia humifusa*; 2n=40) for chromosomal mapping. The analysis of rDNA distribution and CMA (Chromomycin A3) banding across different chromosomes in pitaya and cacti highlights the concept of conserved rDNA. This study provides fundamental insights into cytogenetic markers and their localization across different chromosomes in pitaya and other *Cactaceae* species.

## Introduction

Pitaya, belonging to the *Cactaceae* family, is believed to have diverged from a common ancestor around 35 million years ago. However, significant diversification occurred more recently during the Miocene to Pliocene epochs, approximately 10 to 2.5 million years ago (Arakaki et al., 2011; Khan et al., 2024). This period aligns with a global evolutionary surge in C4 photosynthesis (Arakaki et al., 2011). One hypothesis has suggested that pitaya which is a mostly well-known *Cactaceae* species evolved from a group of cacti and then subsequently adapted and grew in a tropical environment, while another hypothesis suggested that pitaya and other cacti such as *S. grandifloras* evolved independently. The *Cactaceae* family presents challenges due to its varying ploidies and limited genomic data (Hunt et al., 2006; Korotkova et al., 2021). It encompasses a diverse array of plants, with around 100 genera and approximately 1,500 to 1,800 species (Barthlott and Hunt, 1993). Two important species within this family are *H. undatus* and *S. grandiflorus*, which belong to the genera *Hylocereus* and *Selenicereus*, respectively (Tel-Zur et al., 2004). The *Hylocereus* genus comprises approximately 16 species of epiphytic cacti and has sprawling stems that can reach several meters long, with aerial roots that help them attach to trees or other supports (Barthlott and Hunt, 1993). *H. undatus*, commonly known as pitaya or dragon fruit, is a significant tropical fruit crop domesticated from the *Cactaceae* family. It is primarily cultivated as diploid and tetraploid cultivars and tetraploid taxon shares morphological features with diploid (Masashi et al., 2020; Chen et al., 2021; Li et al., 2021; Zheng et al., 2021). The *Selenicereus* genus includes 20 species distributed throughout Mediterranean climates, America and the Caribbean region (Barthlott and Hunt, 1993). *S. grandifloras* is known for its various local names such as night-blooming cactus, large-flowered cactus, sweet-scented cactus, and vanilla cactus which is an important species in the *Cactaceae* (Hecht, 1997). The *Opuntia* genus includes 226 species of cacti commonly known as prickly pears; these species are characterized by flattened, paddle-shaped stems called pads (Castro et al., 2020). *Opuntia humifusa*, known as devil’s tongue, eastern prickly pear, or Indian fig, belongs to the *Opuntia* genus (THE PLANTS DATABASE, see URLs). It is native to regions of the eastern United States, Mississippi, and northeastern Mexico (PLANTS OF THE WORLD, see URLs). All of these plants are vine and succulent native to the tropical and subtropical regions and share similarities such as their capacity to store water in their stems and leaves (Mizrahi and Nerd, 1999).

Chromosome painting by fluorescence *in situ* hybridization (FISH) is an important technique in molecular cytogenetics in plants (Jiang, 2019). This technique is useful for cytogenetic markers mapping, chromosomes identification, polidy determination and karyotyping. However, chromosomal mapping and individual identification are challenging in nonmodel species especially those with large numbers of chromosome or similarly sized chromosome. DNA clone probes such as bacterial artificial chromosome (BAC), rDNA sequences, tandem repeats, and distributed repetitive sequences have traditionally been used for chromosome painting via FISH (Song et al., 2023a, 2023b; Mukai et al., 1993; Jiang et al., 1995; Fransz et al., 1998; Kulikova et al., 2001; Kim et al., 2002; Kato et al., 2004). Due to the limitations of traditional probes, a new class of DNA probes based on low-copy oligonucleotides (so-called single-copy oligo-barcodes) has become popular for FISH experiments (Jiang, 2019; Harun et al., 2023). Oligo-barcodes have been used in an increasing number of plant species for chromosomal identification (Hou et al., 2018; Meng et al., 2018; Braz et al., 2020; Song et al., 2020), mapping (Xin et al., 2018; Bi et al., 2020), karyotyping (Xin et al., 2020; Liu et al., 2020; Braz et al., 2018; Qu et al., 2017; Šimoníková et al., 2019) and rearrangement and translocation (He et al., 2018; Albert et al., 2019; do Vale Martins et al., 2019; Bačovský et al., 2020).

Researchers have explored cytogenetics and evolutionary relationships among plants in the *Cactaceae* family including pitaya, primarily utilizing chromosomes counting, rDNA probes and CMA staining (Lichtenzveig et al., 2000; Castro et al., 2020; Masashi et al., 2020). However, there remains much to uncover, including high-resolution oligo and rDNA mapping, as well as chromosomal identification and karyotyping. Genome sequencing has been completed for pitaya which provides hope for designing and preparing oligo probes (Chen et al., 2021; Li et al., 2021; Zheng et al., 2021). Here, we designed and synthesized thirty-three single-copy oligo-barcodes specific to chromosome regions from the pitaya reference genome. These barcodes were used to map specific oligo sequences on chromosomes. We also applied the same oligo-barcodes to cactus for mapping, chromosomal identification, and karyotyping. Additionally, we mapped two rDNA probes and tandem repeat probes across three *Cactaceae* species. In summary, by performing FISH on these three *Cactaceae* species using oligo-barcodes, rDNA, tandem repeat probes, and CMA, we successfully conducted mapping, chromosome identification, and karyotyping. Our study revealed that the conservation of 45S rDNA has been maintained among pitaya and cactus species since their divergence millions of years ago.

## Materials and methods

### Plant materials and chromosome preparation

*Cactaceae* species, pitaya diploid (2n=2x=22), tetraploid (2n=4x=44), *S. grandiflorus* (2n=2x=22), and *O. humifusa* (2n=2x=40) were used for the experiments. Three diploid species were collected from Huazhong Agricultural University in Wuhan, China, and tetraploid species were obtained from the Chinese Academy of Tropical Agricultural Sciences in Haikou, China. Chromosome preparations for FISH were performed according to reported protocols with minor modifications (Yu et al., 2019). Metaphase chromosome spreads were prepared from the aerial root tips of stem cuttings and good spreads were selected for chromosome counting and other chromosomal analyses. To prepare mitotic metaphase chromosomes root tips were harvested from stems pretreated with a saturated solution of para-dichlorobenzene and a-bromonaphthalene at room temperature (25°C) for 3 h, fixed in Carnoy’s fixative for 12 h, and subsequently stored in 70% ethanol at -20°C until use. An enzyme mixture (1% pectolyase Y23, 2% pectinase, 2% RS, and 4% cellulase Onozuka R-10) was used to digest the root tips for almost 1 h and 30 min at 37°C. Finally, the suspension of cells was dropped onto glass slides and 10 µl of Carnoy’s fixative was used to spread the cells. The chromosomes were stained with DAPI to visualize them clearly in the microscope.

### Development and synthesis of oligo libraries and repetitive sequences

The current study generated 36,944 potential single-copy oligo sequences from the pitaya reference genome (Accession number: PRJNA691451) using the Chorus2 pipeline (Chen et al., 2021). We then synthesized thirty-three oligo-barcodes from the eleven pairs of homologous chromosomes. The design of the oligo-barcodes was performed following the published method with minor modifications (Han et al., 2015). Single-copy oligos with 45 nt length were screened from the reference genome of pitaya (http://pitayagenomic.com/) using the software Chorus2 (Zhang et al., 2021). The RIdeogram (Hao et al., 2020) was used for visualizing the distribution of oligos in the genome. Each oligo-barcode covers a chromosomal region of approximately 0.5 to 1 kb and contains around 1,000 oligos per megabase. The sequences of the oligos are presented in **Supplementary Dataset S1**. The 45S rDNA and 5S rDNA sequences were derived from a sweet orange (*Citrus sinensis*) genome blast. Tandem repeat probes GTR11 and STR3 were obtained by Tandem Repeats Finder (Benson, 1999).

### The labeling of probes

Several primer pairs were added to both ends of each chromosome site-specific oligo and then the oligo-barcode pool was synthesized by the company GENEWIZ (Jiangsu, China). Thirty-three barcodes were selected from the oligo pool using specific primer pairs for PCR amplification. The sequences of the primers used are presented in **Supplementary Dataset S2**. We used the same 45S and 5S probes in the published paper (Song et al., 2023a). Tandem repeat DNAs were obtained by PCR amplifying genomic DNA identified by the Tandem Repeats Finder in silico. Specific primers were used for PCR. The primers for GTR11 and STR3 are shown in **Supplementary Dataset S2**. The sequences of the rDNA and tandem repeat probes are shown in **Supplementary Dataset S3**. GTR11 and STR3 were labeled by PCR (PCR DIG probe DIG synthesis kit, 11636090910; for biotin labeling using Biotin-16-dUTP, 11093070910). Oligo probes were labeled following the method published (Song et al., 2023a).

### FISH and CMA staining

The FISH experiment protocol using oligo and rDNA probes was the same, with a probe concentration of 60 ng/slide. Chromosomal denaturation and hybridization steps were performed according to published procedures (Lan et al., 2016). Approximately 20 µL of hybridization solution containing 1-2 µL of probes was placed on each dried slide and incubated overnight at 37°C. FISH signals were detected according to previously reported protocols (Song et al., 2023a). During the FISH experiment, CMA was used as a reference and DAPI was used for counter-staining. We used *Citrus* (*C. sinensis*) cells as a control during the rDNA FISH experiment (**Supplementary Figure S1**). The FISH images were photographed with a camera (Zeiss Axiocam 506 color, Germany) with ZEN 2 (blue edition) software and then processed with Adobe Photoshop 2020.

### Chromosomal mapping and karyotyping

The actual karyotypes were obtained by measuring the lengths of the long and short arms, as well as the lengths of the CMA bands, using ImageJ (http://rsb.info.nih.gov/ij/) and Adobe Photoshop 2020 software. The distribution of oligo sequences in *H. undatus* was illustrated with the Rideogram (https://github.com/zhangtaolab/Chorus2). The sizes of the 45S and 5S rDNA signals were estimated by measuring their relative lengths in dual-color FISH across 10 metaphase cells using ImageJ software. The estimated relative length is calculated as 100 * (individual length/total length).

## Results

### Chromosome counts

**Figure 1:**
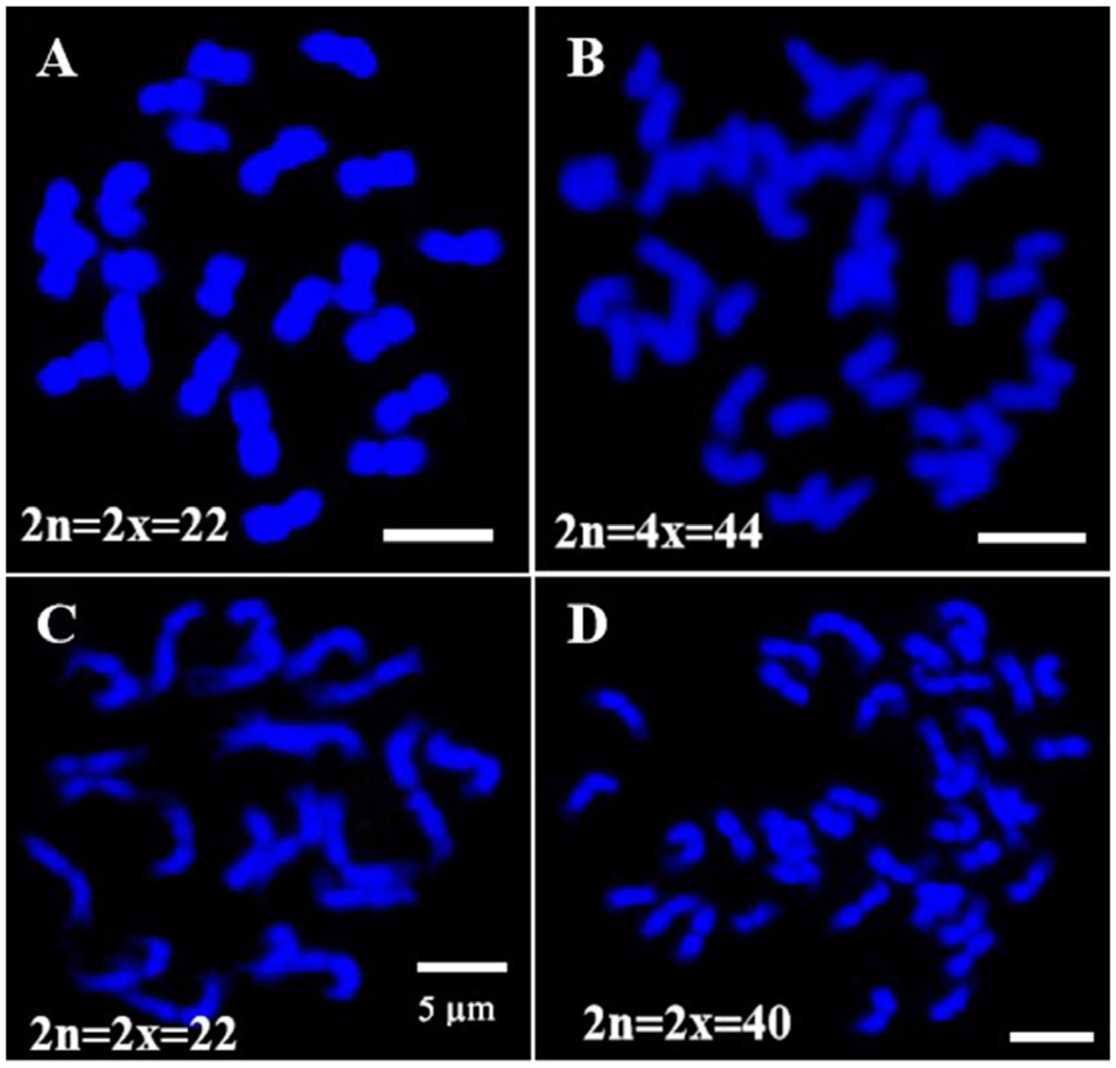
Chromosomes number confirmation of *Cactaceae* species. **(A)** Pitaya diploid (2n=2x=22). **(B)** Pitaya tetraploid (2n=4x=44). **(C)** *S. grandiflorus* (2n=2x=22). **(D)** *O. humifusa* (2n=2x=40). Scale bars=5μm.

Original chromosomes of pitaya and vine cacti were analyzed to identify species and determine their ploidy using FISH. The results showed that the diploid pitaya has 2n=2x=22 chromosomes, while the tetraploid variety has 2n=4x=44 chromosomes (**Figures 1A, B**). For cacti, *S. grandiflorus* has 2n=2x=22 chromosomes, whereas *O. humifusa* has 2n=2x=40 chromosomes (**Figures 1C, D**). We assessed several structural properties of the chromosomes, including chromosomal length (μm), arm length (μm), and arm ratios, which allowed us to construct karyotypes for these species. The average chromosome lengths were moderate: 3.30 ± 0.14 μm for pitaya, 3.27 ± 0.12 μm for *S. grandiflorus*, and 3.05 ± 0.11 μm for *O. humifusa* (**Supplementary Table S1**). All species exhibited symmetrical karyotypes based on centromere position, with notable variation in chromosome size (**Supplementary Table S1**).

### High-resolution oligo map was constructed using oligo-barcodes in pitaya

**Figure 2:**
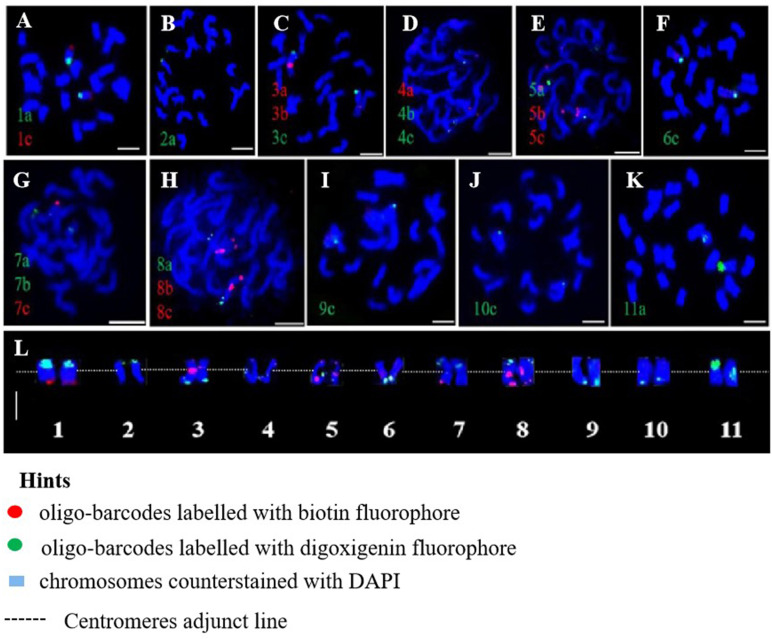
Oligo-barcodes mapping in pitaya and then chromosomes identification using oligo FISH in metaphase cells. **(A–K)** represent chromosomes 1-11 of pitaya respectively with oligo-barcodes modified with red and green fluorophores. **(L)** Chromosomes were digitally separated from **(A–K)** using Adobe Photoshop CS6 ×64 to construct resulted karyotype with oligo-barcodes. Scale bars=5μm.

**Figure 3:**
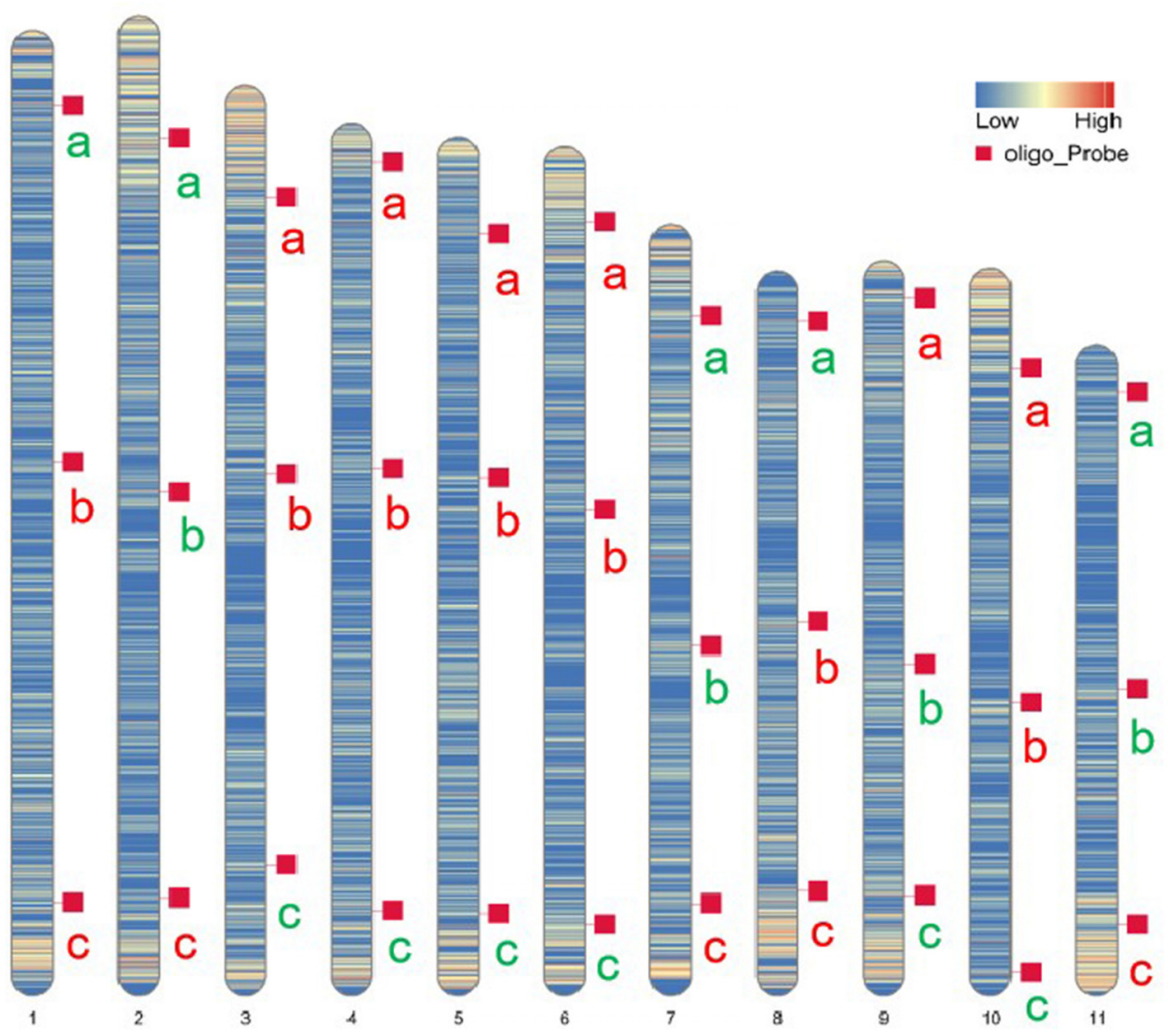
Putative karyotype based on the mapping of oligos onto references genome sequence. Heatmaps represent the density and position of selected oligo-barcodes in pitaya pseduchromosomes. a, b and c denote oligo-barcodes modified with red and green colours by biotin-dUTP and digoxigenin-dUTP antibodies respectively.

Oligo-FISH experiments were conducted using synthesized oligo-barcodes to map high-resolution signals at specific locations on the original chromosomes, allowing for the individual identification of eleven pairs of chromosomes. As expected, each oligo-barcode produced bright FISH signals on one pair of homologous chromosomes (**Figures 2A–K**), while displaying weak noise on other chromosomes (not visible in the figure). Our oligo-FISH experiment successfully mapped nearly all oligo-barcode sites across the homologous chromosomes, with three sites each, although some were missing due to faint signals (1b, 2bc, 6ab, 9ab, 10ab, 11bc). The missing signals could be recovered by redesigning oligo-barcodes to feature longer sequences and fine-tuning the FISH experimental procedure. This mapping was instrumental in identifying specific chromosomes using dual-color FISH. The resulting pattern of oligo-barcodes was digitally constructed after FISH (**Figure 2L**), allowing for a comparison between the constructed and putative karyotypes based on the mapping of oligos onto the reference genome sequence (**Figure 3**).

### Potential oligo-barcodes mapping and chromosomes identification in cactus

**Figure 4:**
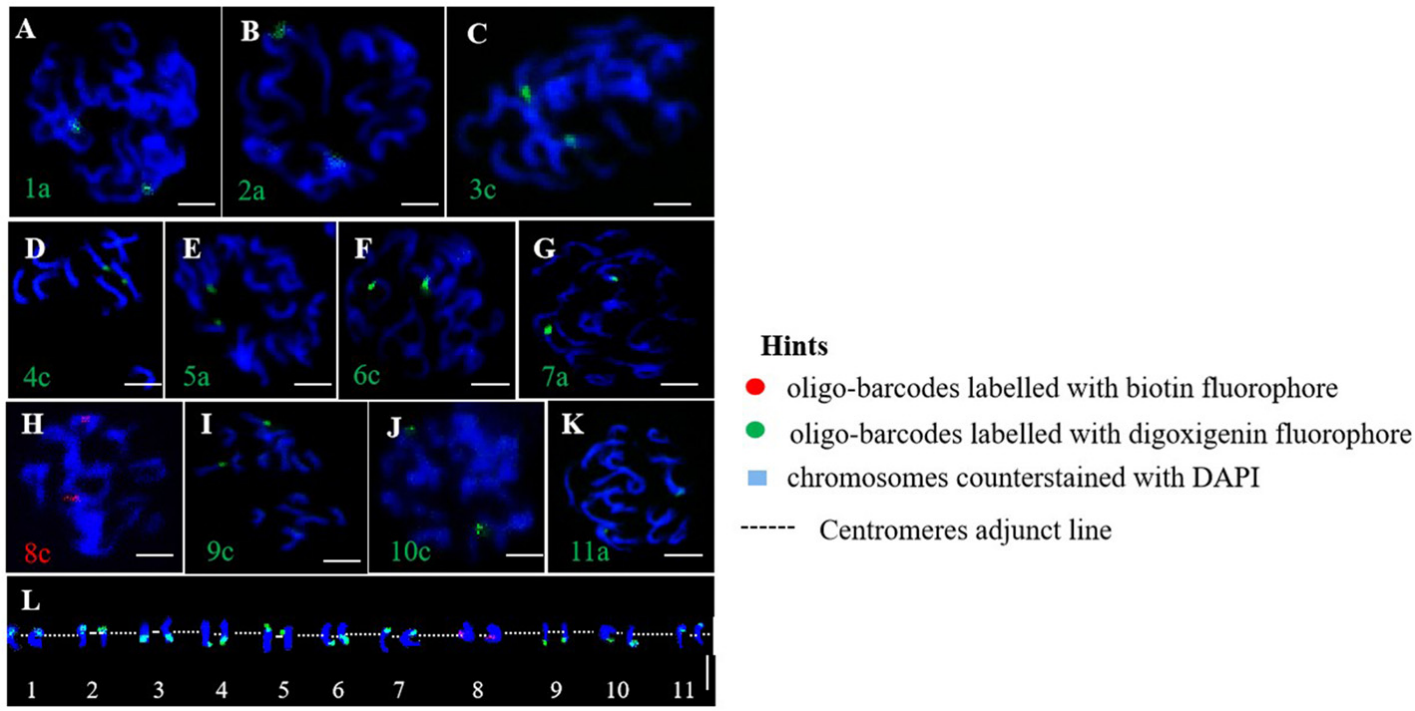
Oligo-barcodes mapping in cactus then chromosomes identification using oligo FISH in metaphase cells. **(A–K)** represent chromosomes 1-11 of cactus respectively with oligo-barcodes modified with red and green fluorophores. **(L)** Chromosomes were digitally separated from **(A–K)** using Adobe Photoshop CS6 ×64 to construct resulted karyotype with oligo-barcodes. Scale bars=5μm.

This study randomly selected eleven oligo barcodes (1a, 2a, 3c, 4c, 5a, 6c, 7a, 8c, 9c, 10c, and 11a) from an oligo probe pool derived from pitaya and applied them to genetically related cactus species for potential testing. We observed bright signals for each oligo probe in the homologous chromosomes of the cacti, which exhibited signal intensities nearly identical to those generated by pitaya (**Figures 4A–K**). A high-resolution oligo map, identification of eleven pairs of homologous chromosomes, and karyotyping were accomplished through the localization of these eleven oligo-barcodes (**Figure 4L**). Our findings suggest that the oligo-barcodes developed from pitaya could serve as universal probes for other *Cactaceae* species; however, the quality and signal intensity of the FISH experiments may vary and should be considered.

### Localization of rDNA cistron and two tandem repeats in *Cactaceae* species

**Figure 5:**
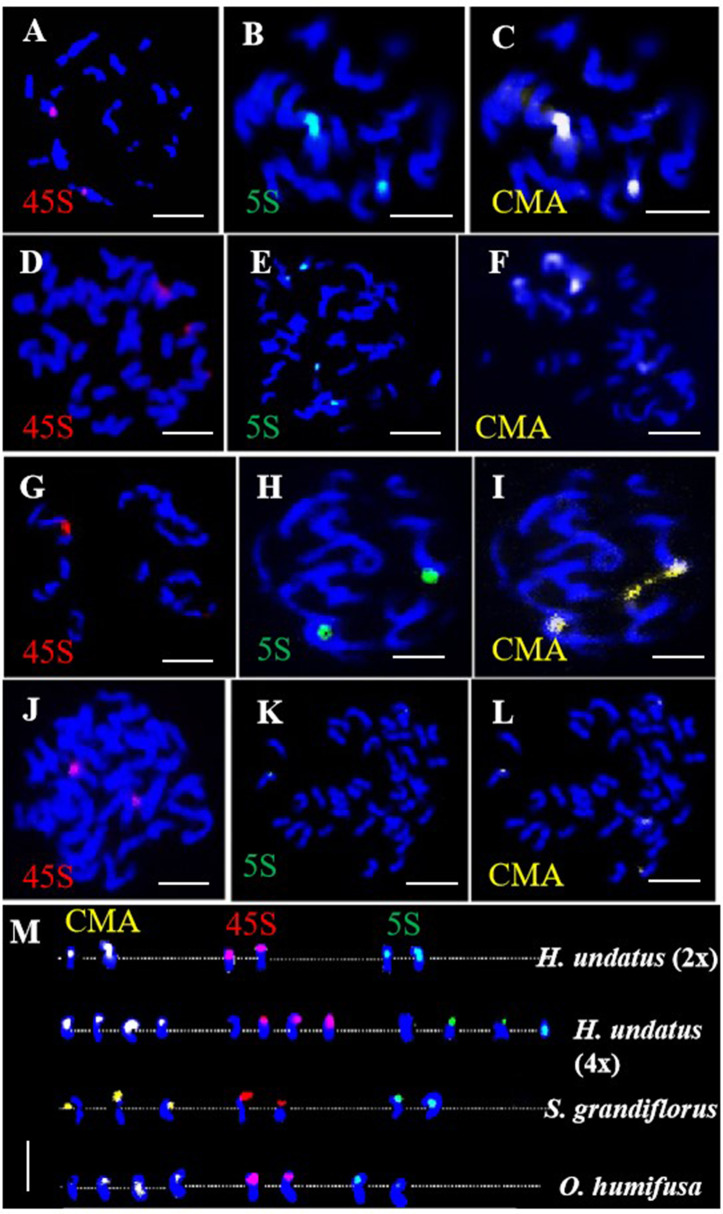
rDNA-FISH of *Cactaceae* species in metaphase cells. **(A–C)** 45S, 5S- rDNA and CMA FISH signals in pitaya diploid respectively. **(D–F)** 45S, 5S- rDNA and CMA FISH signals in pitaya tetraploid respectively. **(G–I)** 45S, 5S- rDNA and CMA FISH signals in *S. grandiflorus* respectively. **(J–L)** 45S, 5S- rDNA and CMA FISH signals in *O. humifusa* respectively. **(M)** Chromosomes were digitally separated from **(A–L)** for rDNA mapping on chromosomes. Scale bars=5μm.

**Figure 6:**
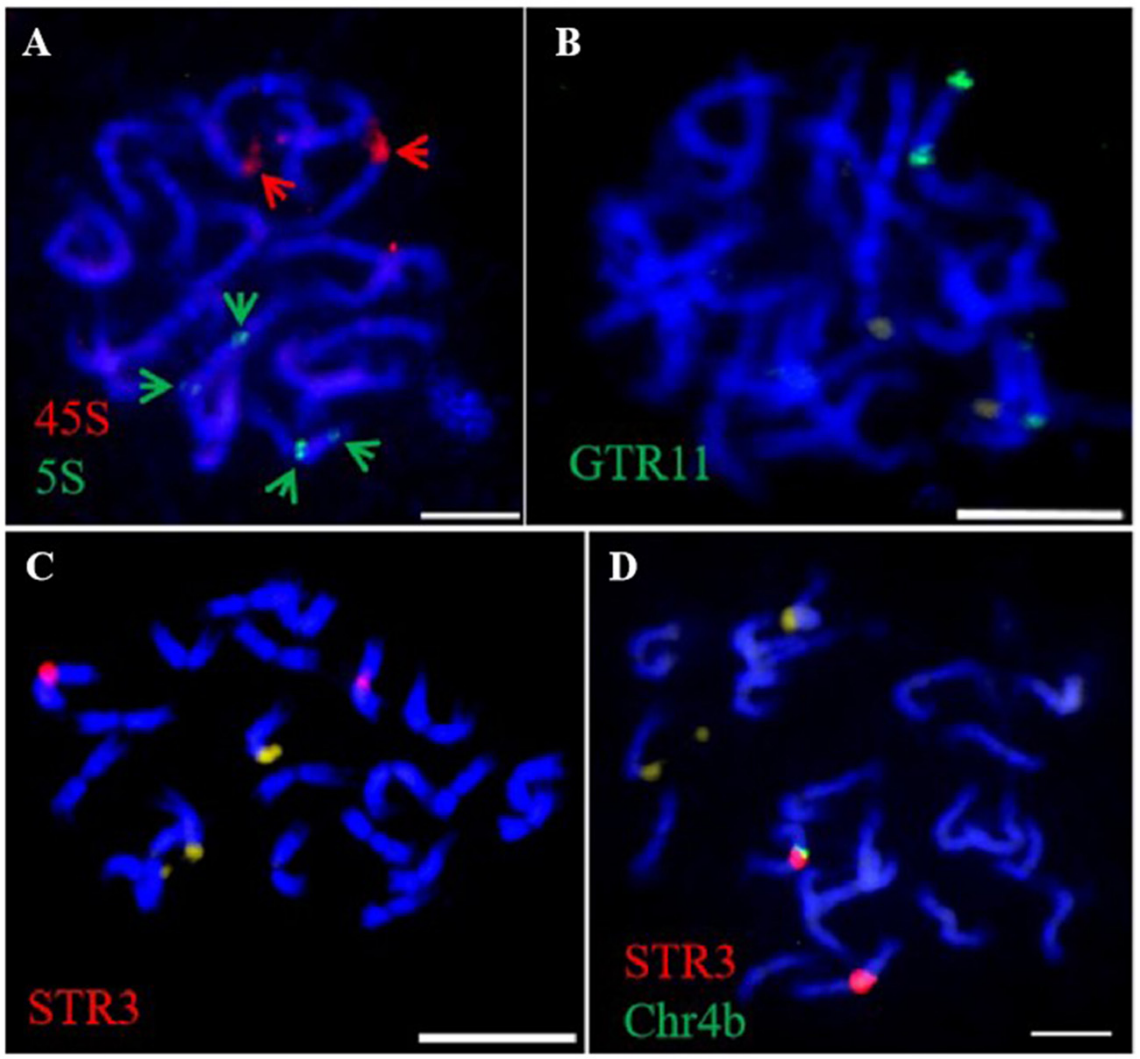
Tandem repeats FISH in pitaya in metaphase cells. **(A)** 45S and 5S- rDNA dual FISH **(B)** GTR11-FISH. **(C)** STR3-FISH. **(D)** STR3 and Chr. 4b oligo -barcodes dual FISH. Scale bars=5μm.

The 45S and 5S rDNA probes were utilized for rDNA-FISH (**Figures 5A–L**). In each diploid species, both 45S and 5S rDNAs were mapped onto two chromosomes, while the 45S rDNA was found to double in the pitaya autotetraploid (**Figure 5M**). rDNA blastn analysis indicated that the 45S rDNA localized on homologous chromosomes 11, and the 5S rDNA localized on homologous chromosomes 7 at subtelomeric positions in pitaya. In the cacti species (*S. grandiflorus* and *O. humifusa*), the 45S and 5S rDNAs were expected to localize at the same site on the same chromosomes of pitaya. However, a standard genome assembly has not yet been reported, preventing the localization of rDNA in these two cactus species. Interestingly, dual-color FISH in pitaya revealed two 45S rDNA signals and four 5S rDNA signals, with two signals positioned centrally and the other two at subtelomeric locations on the chromosomes. The 5S rDNA loci exhibited more heterogeneous profiles, showing two and four loci per diploid genome. The number, localization, and size of rDNA in different *Cactaceae* species are summarized in **Table 1**. The largest 45S rDNA loci were found in *H. undatu*s, while the smallest were in *S. grandiflorus*. The largest 5S rDNA loci were found in *H. undatu*s, while the smallest were in *O. humifusa*. We also screened two tandem repeat probes in pitaya to map additional cytogenetic markers. After labeling, the tandem repeat probes GTR11 and STR3 were employed for FISH. GTR11 localized to the middle and subtelomeric positions of chromosome pair 7, while STR3 was found in the middle position of chromosome 4 (**Figures 6A–D**). Notably, the FISH signals of STR3 were concentrated in the centromeric region of chromosome 4, suggesting that STR3 may be a centromeric tandem repeat. We measured the relative length (Mb) of the CMA banding in ten metaphase cells of diploid and tetraploid pitaya, as well as in *S. grandiflorus* and *O. humifusa* (**Table 1**). Based on CMA banding, most chromosomes in *Cactaceae* species exhibited a D type, while other chromosomes displayed the F type.

**Table 1:** Summary of the molecular cytogenetics of *H. undatus*, *S. grandiflorus* and *O. humifusa*.

Characteristics	*H. undatus* (2x)	*H. undatus* (4x)	*S. grandiflorus*	*O. humifusa*
Chromosomes number	22	44	22	40
Number of signals detected by 45S and	2	4	2	2
Localization	Chr. 11 (subtelomeric)	Chr. 11 (subtelomeric)	×	×
Number of signals detected by 5S and	2 and 4	4	2	2
Localization	Chr. 7 (subtelomeric and middle)	Chr. 7 (subtelomeric)	×	×
Relative length (%) of 45S rDNA	41.30 ± 0.1 32.42 ± 0.2	15.08 ± 0.1 11.65 ± 0.3 16.94 ± 0.2 14.29 ± 0.1	28.45 ± 0.2 17.99 ± 0.1	56.42 ± 0.1 43.59 ± 0.1
Relative length (%) of 5S rDNA	66.67 ± 0.1 23.60 ± 0.1	46.06 ± 0.1 28.43 ± 0.1 29.07 ± 0.3 25.13 ± 0.1	30.08 ± 0.1 21.45 ± 0.3	64.48 ± 0.1 35.53 ± 0.2
Numbers of CMA band	2	4	3	4
CMA band relative length (%)	66.19 ± 0.2 46.86 ± 0.1	83.57 ± 0.3 31.06 ± 0.1 20.22 ± 0.2 29.07 ± 0.1	34.58 ± 0.1 36.73 ± 0.3 89.76 ± 0.1	42.07 ± 0.1 22.88 ± 0.2 18.09 ± 0.1 16.98 ± 0.2

### Molecular ideograms in pitaya and cactus

**Figure 7:**
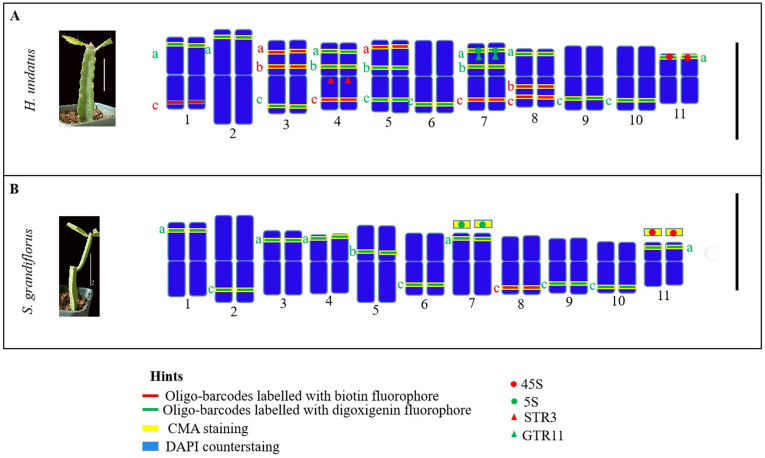
Illustration of ideograms based on oligo-barcodes, rDNA, tandem repeats probes and CMA banding. **(A)** pitaya **(B)** cactus. Scale bars = 10cm and 5µm.

Based on the mapping results of cytogenetic markers, we illustrated their physical positions on the pseudochromosomes of pitaya and cactus species (**Figures 7A, B**). The ideograms depict the locations of oligos and rDNA in pitaya and cacti, as well as the positions of tandem repeats specifically in pitaya. Chromosomes 4 and 7 in pitaya contain a large number of cytogenetic markers. However, illustrating the rDNA markers on specific chromosomes in cactus was not feasible due to the lack of identifiable chromosomal availability.

## Discussion

This study generated thirty-three low-copy oligo-barcodes from eleven homologous chromosomes in pitaya which are short, specific, efficient, and easily labeled. We developed thirty-three oligo-barcodes, of which twenty-two yielded successful FISH results, these twenty-two barcodes have been used for mapping, chromosome identification, and karyotyping in pitaya and its genetically related cactus species which proved that the synthesized oligo probes could be used as universal probes. The oligo-barcodes developed in this study offer several advantages over chromosome painting probes for specific mapping and localizing (Braz et al., 2018, 2020; Meng et al., 2020). However, reliable cytogenetics markars mapping, chromosomes identification and karyotyping are difficult tasks, especially for plants with many chromosomes and limited genomic information including *Cactaceae* (Castro et al., 2020).

This study applied rDNA and tandem repeat probes in pitaya for FISH experiments. While several rDNA studies have been conducted on some *Cactaceae* species (Las Peñas et al., 2009; Moreno et al., 2015; Castro et al., 2020; Masashi et al., 2020, Las Peñas et al., 2013; Tel-Zur et al., 2004) we found that 45S rDNA serves as a valuable cytogenetic marker. Our findings indicate that rDNA sequences in pitaya and related cacti species remain conserved following their divergence (Mizrahi and Nerd, 1999; Garcia et al., 2017; He et al., 2021). We observed 45S rDNA synteny in both pitaya and cacti, which has been preserved in pitaya autotetraploids after genome duplication, challenging misconceptions about the fate of rDNA in polyploidy (Rosselló et al., 2022). The physical mapping of 45S rDNA revealed a conserved pattern, with the number of sites strictly correlated to species ploidy: two sites in diploid species and four sites in tetraploid species (Las Peñas et al., 2009; Moreno et al., 2015; Castro et al., 2016, **Table 1**). All 45S rDNA sites were terminally localized and maybe co-localized with CMA bands, consistent with the most common observations in plants (Lima-de-Faria, 1980; Roa and Guerra, 2012). In contrast, 5S rDNA sites exhibited variability in number and position, occupying proximal and interstitial locations, and occasionally adjacent to 45S rDNA sites, as seen in other *Cactaceae* (Moreno et al., 2015).

The diversity of 5S rDNA sites highlights the significance of structural chromosome rearrangements, such as inversions. This phenomenon may lead to the creation of two sites on the same chromosome arm, as observed in both *Cereus jamacaru* and *Pilosocereus chrysostele* (Castro et al., 2020). It is possible that a breakpoint occurred within the original 5S rDNA site an event potentially favored by transposable element (TE) activity. Following an inversion, some copies of 5S rDNA could have been inserted, creating a new site while retaining copies at the original site. Such events have been suggested for various plant groups, including unrelated species of *Orchidaceae* (Moraes et al., 2012; 2017; Lee et al., 2017). In this sense, the evolution of 5S rDNA sites in cacti contrasts with the evolution of 45S rDNA, with 5S being more variable than 45S, which is the opposite of the commonly accepted hypothesis that position and number of 5S rDNA loci in plants are usually more conserved than those of 45S rDNA loci (Roa and Guerra, 2012, 2015).

It is noteworthy that the FISH signals of STR3 are localized in the centromeric region of chromosome 4, suggesting that STR3 may represent a centromeric tandem repeat. Additionally, centromeric repeats are highly conserved within the karyotypes. However, the STR3 repeat is only detected in a single chromosome pair in pitaya, and tandem repeats are not conserved between chromosomes may be due to evolutionary pressures, and its genetics makeup in pitaya (Ma et al., 2023). The presence of terminal CMA bands observed in this study appears to be a common characteristic among plant species (Moreno et al., 2015; Las Peñas et al., 2013, 2009). Variation in heterochromatic bands has long been utilized for karyotypic characterization among species, such as in *Orchidacea*e (Moraes et al., 2017, 2016; Koehler et al., 2008). The CMA band pattern has proven to be taxonomically informative in cacti, providing valuable chromosome markers within the stable karyotypes typical of the *Cactaceae* family.

## Conclusions

We developed thirty-three oligo probes from the pitaya reference genome for mapping in both pitaya and cactus, as well as for chromosome identification. Ideograms of pitaya and cactus were illustrated based on oligo-barcodes, rDNA, and tandem probes. The cactus ideogram was constructed for comparison with pitaya, revealing that both species exhibit symmetrical karyotypes. Analysis of the distribution of 45S, 5S rDNA and CMA across various *Cactaceae* species highlighted 45S conservation, while the movement of 5S rDNA in pitaya may facilitate the creation of additional 5S rDNA sites throughout the genome. This research utilizes mapped molecular cytogenetic markers in pitaya and cacti, providing valuable insights into their cytogenomic structure and evolutionary divergence from a common ancestor.

## Glossary

Karyotyping: A laboratory technique used to analyze an individual’s chromosomes by arranging and staining them to create a visual representation known as a karyotype. This process allows for the identification of the number, size, and shape of chromosomes.

rDNA Probe: A molecular cytogenetics tool designed to detect specific DNA sequences related to ribosomal DNA (rDNA). These probes are typically labeled with a fluorescent marker, enabling visualization and identification of rDNA presence in various samples, such as tissues or cells.

Homologous Chromosomes: Pairs of chromosomes that contain the same genes in the same order, with one chromosome inherited from each parent within the same species.

Cytogenetic Mapping: The process of determining the physical locations of cytogenetic markers such as oligo sequences, tandem repeats, telomere repeats, and rDNA along the chromosomes.

Evolution: The study of changes in genetic sequences and the resulting modifications in biological macromolecules, including oligos, proteins, and rRNA, over time.

## URLs

The Plants Database (National Plant Data Center), http://plants.usda.gov; POWO (Plants of the World Online), http://www.plantsoftheworldonline.org.

## Data Availability

The datasets presented in this study can be found in online repositories. The names of the repository/repositories and accession number(s) can be found in the article/**Supplementary Material**.

## Supplementary material

Supplementary Figure 1 45S rDNA FISH and CMA banding in sweet orange (*Citrus sinensis*) used as positive control during rDNA FISH experiment in pitaya. **(A)** 45S rDNA FISH in metaphase cell. Arrows show 45S rDNA FISH signals in *Citrus*. **(B)** CMA staining in metaphase cell. Scale bars=5μm.

Supplementary Table 1 Comparative karyotyping among *H. undatus*, *S. grandiflorus* and *O. humifusa*.

Supplementary Dataset 1 All oligo sequences in pitaya reference genome.

Supplementary Dataset 2 List of primers used in this study for synthesizing oligo-barcodes.

Supplementary Dataset 3 Sequences of two rDNAs and two tandem repeats.
