# Supplementary material for: Comprehensive mapping of molecular cytogenetic markers in pitaya (Hylocereus undatus) and related species
Source: Front Plant Sci. 2024 Dec 6;15:1493776. doi: 10.3389/fpls.2024.1493776 (PMC11662977; doi:10.3389/fpls.2024.1493776)
Supplement: Supplementary Figure 1 — 45S rDNA FISH and CMA banding in sweet orange (Citrus sinensis) used as positive control during rDNA FISH experiment in pitaya. (A) 45S rDNA FISH in metaphase cell. Arrows show 45S rDNA FISH signals in Citrus. (B) CMA staining in metaphase cell. Scale bars=5μm. [file DataSheet1.zip › Supplementary Table 1.docx]

**Table S1|** Comparative karyotyping among *H. undatus*, *S. grandiflorus* and *O. humifusa*.

|  | ***H. undatus*** | | | ***S. grandiflorus*** | | | | ***O. Humifusa*** | | |
| --- | --- | --- | --- | --- | --- | --- | --- | --- | --- | --- |
| **Chrs. Number** | **Long arm**  **(μm)** | **Short arm (μm)** | **Total length (μm)** | | **Long arm (μm)** | **Short arm (μm)** | **Total length (μm)** | **Long arm (μm)** | **Short arm (μm)** | **Total length (μm)** |
| 1 | 2.0±0.1 | 1.85±0.1 | 3.85±0.1 | | 2.53±0.1 | 1.6±0.1 | 4.13±0.1 | 2.14±0.1 | 1.91±0.1 | 4.05±0.1 |
| 2 | 2.76±0.1 | 1.84±0.2 | 4.60±0.2 | | 2.52±0.1 | 2.08±0.1 | 4.60±0.1 | 2.54±0.1 | 1.91±0.1 | 4.45±0.1 |
| 3 | 2.10±0.1 | 1.31±0.1 | 3.41±0.1 | | 1.86±0.1 | 1.82±0.1 | 3.68±0.1 | 1.84±0.1 | 1.63±0.1 | 3.47±0.1 |
| 4 | 1.71±0.1 | 1.63±0.2 | 3.34±0.2 | | 1.97±0.1 | 1.64±0.1 | 3.61±0.2 | 1.62±0.1 | 1.41±0.1 | 3.03±0.1 |
| 5 | 1.83±0.1 | 1.66±0.1 | 3.49±0.1 | | 2.34±0.1 | 1.44±0.4 | 3.78±0.1 | 2.28±0.1 | 1.76±0.1 | 4.04±0.1 |
| 6 | 1.71±0.4 | 1.62±0.2 | 3.33±0.3 | | 1.70±0.3 | 1.62±0.1 | 3.32±0.1 | 1.99±0.1 | 1.56±0.1 | 3.55±0.1 |
| 7 | 1.59±0.1 | 1.58±0.1 | 3.17±0.1 | | 1.92±0.1 | 1.41±0.1 | 3.33±0.1 | 1.62±0.1 | 1.26±0.1 | 2.88±0.1 |
| 8 | 1.46±0.1 | 1.32±0.1 | 2.78±0.1 | | 1.51±0.1 | 1.42±0.1 | 2.93±0.1 | 1.02±0.1 | 0.97±0.1 | 1.99±0.1 |
| 9 | 1.68±0.1 | 1.33±0.1 | 3.01±0.1 | | 1.34±0.1 | 1.12±0.1 | 2.46±0.1 | 1.74±0.1 | 1.64±0.1 | 3.38±0.1 |
| 10 | 1.76±0.1 | 1.13±0.1 | 2.89±0.1 | | 1.13±0.1 | 1.09±0.3 | 2.22±0.3 | 1.89±0.1 | 1.83±0.1 | 3.72±0.1 |
| 11 | 1.19±0.1 | 1.19±0.2 | 2.38±0.2 | | 1.10±0.1 | 0.81±0.1 | 1.91±0.1 | 1.86±0.1 | 1.30±0.1 | 3.16±0.1 |
| 12 |  |  |  | |  |  |  | 1.91±0.1 | 1.20±0.1 | 3.11±0.1 |
| 13 |  |  |  | |  |  |  | 1.53±0.2 | 1.27±0.1 | 2.80±0.1 |
| 14 |  |  |  | |  |  |  | 1.67±0.1 | 1.27±0.2 | 2.94±0.1 |
| 15 |  |  |  | |  |  |  | 2.03±0.1 | 1.94±0.2 | 3.97±0.1 |
| 16 |  |  |  | |  |  |  | 1.79±0.3 | 1.46±0.1 | 3.25±0.2 |
| 17 |  |  |  | |  |  |  | 1.57±0.1 | 1.15±0.2 | 2.72±0.1 |
| 18 |  |  |  | |  |  |  | 1.28±0.1 | 1.18±0.1 | 2.46±0.1 |
| 19 |  |  |  | |  |  |  | 1.47±0.1 | 1.00±0.1 | 2.47±0.1 |
| 20 |  |  |  | |  |  |  | 1.02±0.1 | 0.97±0.2 | 1.99±0.2 |
